# Letter to the editor about “Pediatric trichinosis: A case report”

**DOI:** 10.1016/j.ijscr.2024.110731

**Published:** 2024-12-10

**Authors:** Jean Dupouy-Camet, Fabrizio Bruschi, Edoardo Pozio

**Affiliations:** a Paris Cité Medical Faculty, French Veterinary Academy, Paris, France; b University of Pisa, Pisa, Italy; c Department of Infectious Diseases, Istituto Superiore di Sanita, Rome, Italy

**Keywords:** Trichinellosis, *Trichinella*, Clinical aspects

We are a bit dubitative about the case of pediatric trichinellosis (the term trichinosis used by the authors is now obsolete…) reported in the November 2024 issue of Int J Surg Case Rep [1]. As experts in the field, we found many drawbacks in this report [2,3]. According to the authors, the child developed clinical signs compatible with trichinellosis after family pork consumption. “*None of them had developed these symptoms except this child*” but the only symptoms reported in the child were “*fever, cough, weight loss, night sweating, loss of appetite, pressure effects, or bowel, bladder, joints or nervous system involvement*”. These signs are not at all specific to *Trichinella* infection! No muscular pain, no facial oedema, were mentioned. In addition, at presentation, there was no fever and just a moderate increased eosinophilia (900 eosinophils/microl). Serological tests for *Trichinella*, filariasis and *Toxocara*, blood search for microfilariasis and stool examination were not performed. According to the authors, the diagnosis is only based on a muscular biopsy showing “*calcified larvae and encysted Trichinella larvae (nurse cell) in skeletal muscle cells*”. In our opinion, the figure shows a granuloma, but we do not see any nurse cell, there is no modified muscular fiber and no collagen capsule. There is a probable section of a nematode worm, but it is not a *Trichinella* larva. In the discussion, the authors are only comparing this clinical case with a previous Ethiopian case [4]. “*Similar to our case, a 14-year-old male patient from West Arsi Zone, Oromia Region, Ethiopia, presented with a complaint of asymmetric right thigh enlargement of one month duration for which biopsy was done and diagnosed to have trichinosis*”. In this case, the biopsy shows indisputable *Trichinella* larvae.

In conclusion, the case reported by Kindie et al. cannot be defined as a case of trichinellosis. A lymphatic filariasis such as *Wuchereria* could have been responsible for this neck enlargement, but ultrasounds did not evidence any lymph node enlargement, and the blood eosinophile counts were not so high as expected in such helminthiasis. Furthermore, *Wuchereria* nematodes do not, usually, migrate in the muscles. On the contrary, *Toxocara* larvae can migrate in the muscles and might be responsible for this swelling, which could have been cured by the albendazole treatment prescribed to the child. Unfortunately, no search for *Toxocara* antibodies was performed to confirm this hypothesis. Ethiopia is, without any doubt, a country where nematodes of the genus *Trichinella* are circulating in wildlife [5], but this case does not fit with the clinical signs of trichinellosis.

## Author contribution

JDC, FB & EP contributed equally to the writing of the letter. They are members of the International commission on trichinellosis.

## Consent

Not applicable.

## Ethical approval

Not applicable.

## Guarantor

Jean Dupouy-Camet.

## Research registration number

Not applicable.

## Funding

No funding.

## Conflict of interest statement

None of the authors have conflict of interest.
